# Silent pulmonary thromboembolism in neurosurgery patients: Report of 2 cases and literature review

**DOI:** 10.1097/MD.0000000000004589

**Published:** 2016-08-19

**Authors:** Rui Tian, Jun Gao, Alof Chen, Xinjie Bao, Jian Guan, Ming Feng, Yongning Li, Wenbin Ma, Zuyuan Ren, Renzhi Wang, Junji Wei

**Affiliations:** Department of Neurosurgery, Peking Union Medical College Hospital, Chinese Academy of Medical Sciences and Peking Union Medical College, Beijing, China.

**Keywords:** asymptomatic pulmonary thromboembolism, deep vein thrombosis, neurosurgery, silent pulmonary thromboembolism

## Abstract

**Background:**

The requirement of postoperative bedridden and immobilization renders neurosurgical patients with higher risk of deep vein thrombosis (DVT), then more vulnerable for pulmonary thromboembolism (PTE). But silent pulmonary thromboembolism (SPTE) can be the very early stage of any typical form of PTE, its diagnosis and management is therefore critical in neurosurgical departments. However, to date, perioperative SPTE has not been attached with enough attention.

**Methods:**

Here, we report 2 cases of perioperative SPTE in the Department of Neurosurgery, Peking Union Medical College Hospital, Beijing, China. Clinical data of 2 cases was collected and analyzed. Both patients were screened by quantitative D-dimer assay and lower limbs ultrasonography, while diagnoses were made according to computed tomographic pulmonary angiography (CTPA). Therapeutic medications include heparin, low molecular weight heparin, followed by long-term anticoagulation with oral warfarin. Both cases showed significantly elevated D-dimer before and after onset of SPTE. But in 1 case, ultrasonography reported negative venous thromboembolism. CTPA confirmed all diagnosis of SPTE. Repeated CTPA after anticoagulant therapy identified therapeutic efficacy. And during the follow-up period of 5 or 6 years, both patients acquired full recovery without clinical complications.

**Results:**

Significant decline of D-dimer was observed after the comprehensive management of SPTE (case 1: preop vs postop 573 vs 50 μg/L; case 2: preop vs postop 246 vs 50 μg/L). Ultrasonography was used for suspicious of DVT, while CTPA was used for confirming SPTE diagnosis.

**Conclusion:**

Clinicians should be aware of the importance of early recognition of SPTE. Effective management of risk factors of hyper-coagulation state should be the key to prophylaxis. And routine monitor of D-dimer as well as regular check of lower limbs ultrasonography should be standardized and included in guidelines of neurosurgical patient management.

## 1 Introduction

In the field of neurosurgery, pulmonary thromboembolism (PTE) has been a challenge in perioperative patient management. PTE is the obstruction of the pulmonary arteries by thrombus derived from the venous system or right heart, clinically featured with compromised pulmonary circulation and respiratory failure. In clinic, 80% to 90% of the PTE patients have obvious symptoms. Whereas silent pulmonary thromboembolism (SPTE) refers to those diagnosed by examination, but have no typical presentation. In classification, SPTE is the very early stage of any typical class of PTE. It is also a life-saving step of the “DVT-SPTE-PTE-death” chain.

The direct criminal of SPTE attack is deep vein thrombosis (DVT). In the setting of neurosurgery, the requirement of postoperative bedridden and immobilization of neurosurgical patients renders them with higher risk of DVT. But in the past decades, SPTE has been more or less ignored by clinicians due to its silent manifestation and limit of old detection method. In recent years, screening studies report its high prevalence under computed tomographic pulmonary angiography (CTPA)^[1]^ and it accounts for 3% of all perioperative death in neurosurgery. Thus, as a critical and life-saving step of the “DVT-SPTE-PTE-death” chain, management of SPTE in neurosurgical department can never be neglected. However, to date, little attention has been attached on the issue and no guideline has been published.

Here, we reported 2 representative (but not all circumstances) SPTE cases of early recognition and perioperative management of SPTE, either with positive D-dimer and negative ultrasonography or with positive ultrasonography and negative D-dimer. A comprehensive review of our current understanding of SPTE is provided, with a focus on SPTE in the setting of neurosurgery.

## 2 Case report

### 2.1 Case 1

A 48-year-old female was presented with dizziness for 2 months. No remarkable findings in medical history, neurological and physical examinations, and ECG. Head MRI supported the diagnosis of left side frontal parasagittal meningioma. A transcranial meningioma resection was therefore operated. The operation duration was 6 hours, with a total of 3000 mL of blood loss and 1000 mL of autotransfusion, and the patient returned to neurosurgical intensive care unit (NICU). Graded compression stockings were used for the prophylaxis of thrombotic events. Postoperative neurological examination revealed right-sided hemiplegia with muscle strength graded 3/5 in upper limb and 0/5 in lower limb.

**Figure 1:**
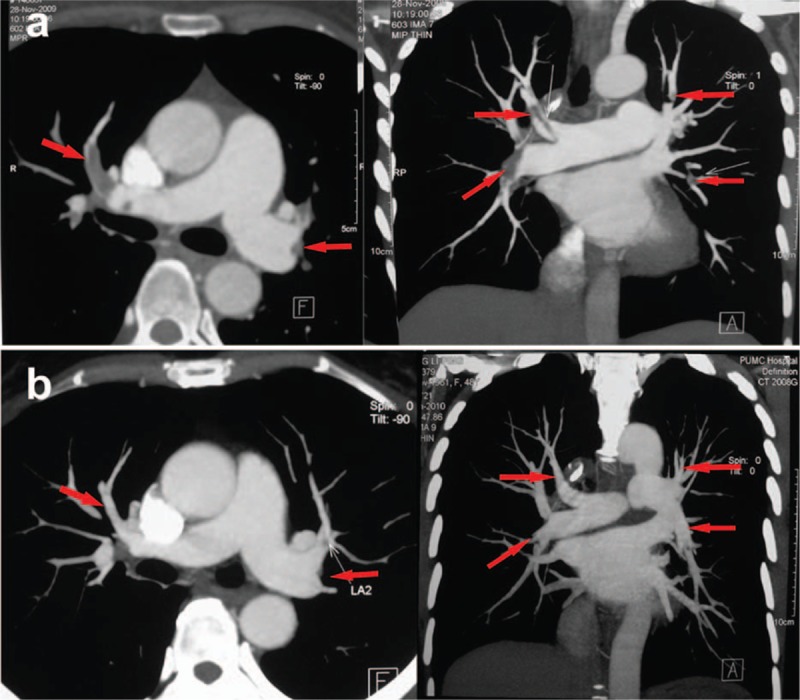
Change of the volume of thromboembolism before and after anticoagulation in case 1. (A) CTPA revealed multiple PTE in the pulmonary arteries of the left anterior superior lobe, left anterior inferior-medial lobe, right medial-middle lobe, and right inferior lobe. (B) CTPA showed that the PTE in the left anterior superior lobe, left anterior inferior-medial lobe, right medial-middle lobe, and right inferior lobe was dissolved. CTPA = computed tomographic pulmonary angiography, PTE = pulmonary thromboembolism.

**Figure 2:**
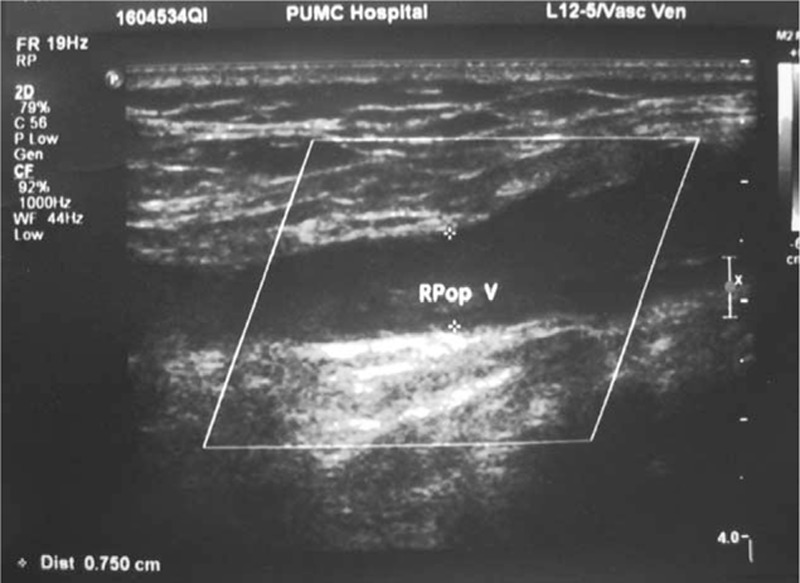
Lower limbs ultrasonography examination revealed thrombosis in the right popliteal vein in case 1.

Six days after surgery, the patient complained of severe headache. Emergency head CT revealed local brain edema and intracranial hematoma, indicating for urgent decompressive craniectomy and hematoma debridement. After 4 hours’ reoperation, headache was relieved but right-sided hemiplegia still existed. Slight abnormality of vital sign was observed, including fluctuation of heart rate between 80 and 100 bpm, a mild decrease of blood pressure to 100/60 mm Hg, and notably, a decrease in finger oxygen saturation from 100% to 98%. Arterial blood gas analysis revealed respiratory alkalosis (pH 7.451, PO_2_ 66.1 mm Hg, CO_2_ 34.8 mm Hg). Further test on coagulation reported D-dimer 573 μg/L (normal range <420 μg/L). Emergency CTPA revealed multiple embolisms in pulmonary arteries of the left anterior superior lobe, left lingual lobe, left anterior inferior-medial lobe, right medial-middle lobe, and right inferior lobe (Fig. 1A), although bed-side ultrasonography that night for DVT in bilateral lower limbs was negative. SPTE was diagnosed according to the patient's CTPA image and “silent” manifestation, though ultrasonography found no DVT. Upon diagnosis, 12,500 units of unfractionated heparin were diluted as 250 U/mL solution and pumped at 2 mL/hour following 1st bolus of 9 mL. Coagulation was monitored every 2 hours to adjust bump rate to maintain APTT within 50 to 70 seconds. Further adjustment of 3 mg qd oral warfarin was added 3 days later (9 days after 1st surgery) for 3 to 5 days’ overlap, before discontinuation of unfractionated heparin. Dynamic adjustment of warfarin dosage must be highlighted to maintain INR between 1.8 and 2.5. And 2 weeks after SPTE diagnosis (20 days after 1st surgery), repeated lower limbs ultrasonography showed DVT in right popliteal vein (Fig. 2). The patient then discharged with 6 mg/7.5 mg qod oral warfarin and was followed on clinic with regular monitor on D-dimer and ultrasonography every 2 weeks.

One month after discharge (on the 2nd follow-up), the patient presented with improved right-side muscle strength, upper limb graded 5/5, and lower limb graded 4/5. Ultrasonography revealed a shrinkage in the volume of DVT. CTPA showed most of the thrombosis dissolved (Fig. 1B), and D-dimer decreased to 150 μg/L (normal range <420 μg/L). After 6 years’ follow-up, the patient has no complaints of any discomfort or adverse event.

### 2.2 Case 2

A 69-year-old hypertensive male was admitted with limb weakness, acroanesthesia, and walking limitation for 5 months. Neurological examination revealed complete sensory loss below C6–7 level, with decreased muscle strength (distal left arm graded 5-/5, proximal right arm graded 4/5, proximal left leg graded 3/5, distal left leg graded 4/5, proximal right leg graded 4/5, and distal right leg graded 5-/5) and bilateral positive Chaddock sign. Extra-spinal subdural meningioma at C6–7 was diagnosis with spinal MRI. A spinal meningioma resection surgery was performed under general anesthesia with cervical posteromedial approach, and the patient returned to NICU. Graded compression stockings were used for the prophylaxis of thrombotic events. Postoperative neurological examination revealed further decreased muscle strength of 3/5 in both lower limbs.

**Figure 3:**
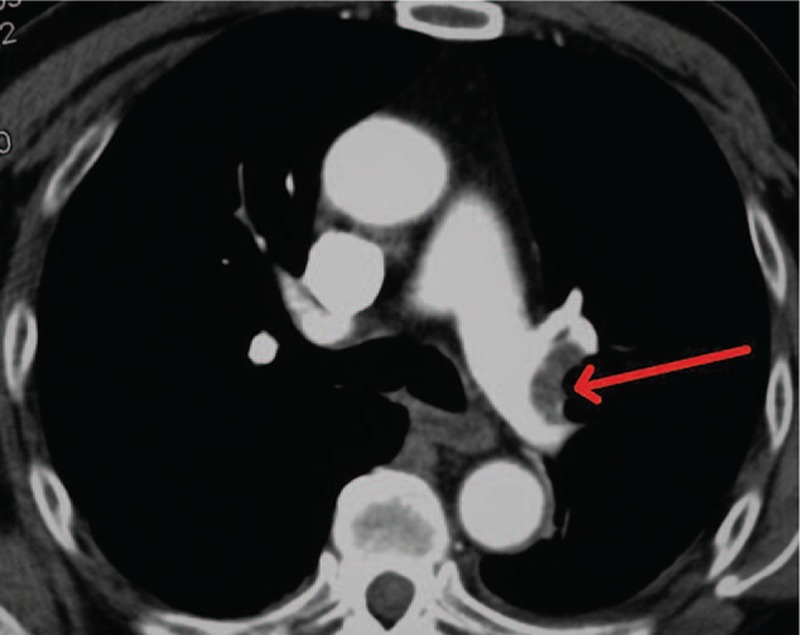
CTPA confirmed the diagnosis of PTE in the left superior and left inferior pulmonary arteries in case 2. CTPA = computed tomographic pulmonary angiography, PTE = pulmonary thromboembolism.

Nine days postoperation, the patient complained of sudden heart palpitation. The NICU monitor also reported intermittent tachycardia at 120 bpm, though with normal blood pressure and oxygen saturation, and no edema was found. Physical examination revealed increased respiration rate at 27 times/minute. Myocardial enzymes were negative. Bed-side electrocardiograph revealed slight lowering of T waves in V3 and V4 leads. Arterial blood gas analysis showed pH 7.483, PCO_2_ 3.8 mm Hg, and PO_2_ 89.4 mm Hg. Normal PT/APTT with D-dimer 246 mg/L. Considering that he had been on regular Terazosin for hypertension control, and tachycardia is one of the common side-effects of Terazosin, the medication was discontinued. Later in the mid-night, the patient's vital signs returned normal. The next day, lower limbs ultrasonography examination showed extensive thrombosis in the right distal common femoral vein, superficial femoral vein, gluteal vein, and posterior tibial vein. Furthermore, CTPA confirmed SPTE in the left superior and inferior lobar pulmonary arteries (Fig. 3). Inferior vena cava filter placement was performed under local anesthesia. After the operation, 0.6 mL q12h low molecular weight heparin was administered, and further adjustments of oral warfarin was added 2 days later at an initial dose of 2.25 mgqd. Coagulation and D-dimer were monitored to adjust warfarin, for which INR was aimed within 1.8 to 2.5. When D-dimer decreased to 50 μg/L, the patient discharged with oral warfarin 3 mg qd (18 days after 1st surgery).

**Figure 4:**
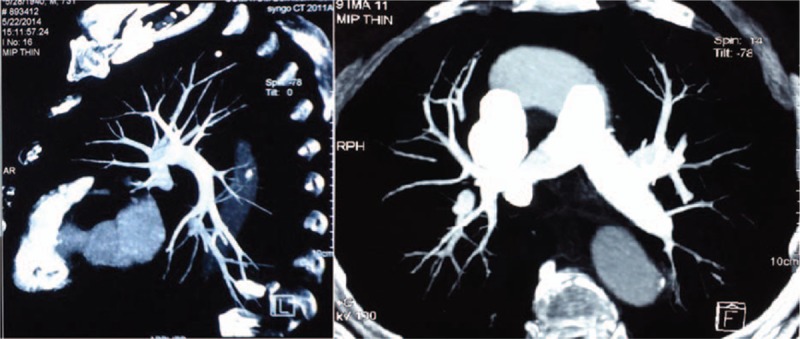
CTPA showed that most of the PTE in the left superior and left inferior pulmonary arteries was dissolved in case 2. CTPA = computed tomographic pulmonary angiography, PTE = pulmonary thromboembolism.

Two months postdischarge (on the 4th follow-up), the patient presented with improved muscle strength without walking difficulty. Ultrasonography revealed decreased volume of DVT. CTPA showed most of the thrombosis was dissolved (Fig. 4) and D-dimer decreased to 30 μg/L (normal range <420 μg/L). During 5 years’ regular follow-up, the patient has no complaints of any discomfort or adverse event.

## 3 Discussion

Effective management of SPTE-associated risk factors is the key to prophylaxis. With regards to hospitalized patients, the population at the risk of venous thromboembolism (VTE) is related with past history of VTE, elderly (>75 year-old), near-term surgery or trauma, immobility or paresis, obesity with BMI >30, central venous catheterization, thrombophilic states, varicose veins, estrogen therapy, or stroke.^[2]^ For stroke, it is noted that presence of preexisting neurological deficit in the form of hemiplegia or hemiparesis appears to have strong relationship with DVT.^[3]^ Coexisting heart diseases also attribute to the prevalence of SPTE.^[4,5]^ Other independent predictors of VTE include: male gender, right lower limbs, unprovoked DVT,^[6]^ ventilator dependence, malignancy, and surgical procedures.^[7]^ For neurosurgical procedures, it was shown that patients underwent cranial procedures are significantly more likely to suffer VTE than those underwent spinal procedures.^[8]^ Other predictors that affect the incidence of SPTE include proximal location of DVT,^[9,10]^ long-term steroid use, sepsis,^[7]^ as well as brain tumor.

Brain tumor patients have been observed to be at increased risk of developing VTE than patients with tumors of other sites.^[11]^ For those who received craniotomy for brain tumor,^[12]^ especially with glioma (15%) and meningioma (7.1%), higher incidence of DVT makes them more vulnerable for PTE.^[3]^ Related risk factors may include solid tumor, use of osmotherapeutic drugs, hormone replacement therapy, elongated surgical time span (>4 hours), intraoperative bleeding, chemotherapy, and most of all, the requirement of postoperative bedridden and immobilization. The underlying mechanism of why brain tumor is associated with hyper-coagulation is mainly about cytokines metabolism and hypoxia microenvironment.^[13–15]^ An epidemiology study based on the National Blood Clot Alliance revealed that poor awareness of VTE has been raised among cancer patients, and more effective communication between patients and physicians about thromboprophylaxis is of great importance.^[16]^ On the other hand, VTE can be the earliest sign of tumor development, but early screening of occult tumors in VTE patients is disputable. A randomized controlled clinical trial assessing the screening strategy by abdomen-pelvis computed tomography suggested poor relevance between 1st episode of unprovoked VTE and occult tumor.^[17]^ Thus, the relationship works in both ways: patients with brain tumor are at increased risk of VTE, meanwhile VTE may be the earliest sign of tumor.

Up to now, there is no consensus on choice of anticoagulant for SPTE. A randomized study finished by the EINSTEIN investigators on VTE patients with symptomatic PTE^[18]^ suggested rivaroxaban as a friendly single-drug treatment, either by short-term or by long-term. In contrast to enoxaparin plus a vitamin K antagonist as placebo, rivaroxaban can ameliorate the benefit-to-risk profile of anticoagulation treatment. As for choice of classic medications, most randomized controlled trials showed no significant difference between low molecular weight heparin and unfractionated heparin. Optimal duration of anticoagulation treatment has been controversial before a randomized, double-blind clinical trial was recently released. The researchers compared the outcomes of 6-month initial anticoagulation (nonrandomized administration on a vitamin K antagonist) with additional 18-month anticoagulation (randomized administration on warfarin) after 1st episode of unprovoked PTE. In comparison with placebo, the additional 18-month warfarin administration abridged the recurrence of VTE or major bleeding, even though the advantage may not be preserved after discontinuation of warfarin.^[19]^ And after discontinuation of anticoagulation in unprovoked VTE patients, low-dose aspirin can be used as prophylaxis against recurrence, no increased risk of major bleeding is observed.^[20]^ For pituitary tumor patients with Cushing disease, hyper-coagulation state featured with higher incidence of VTE is a common complication, and deserves active thromboprophylaxis. Nadroparin 5700 U, dalteparin 5000 U, or enoxaparin 40 mg sc once daily is recommended until optimal regimen will be yielded from prospective studies.^[21]^

Although over one third DVT patients are complicated with SPTE,^[1]^ the necessity of SPTE routine screening among DVT patients remains controversial. There are studies believing routine screening with CTPA would increase the risks of radiation-associated malignancy, contrast-induced nephropathy, and the result of screening will not change DVT patient management.^[22]^ But these should not be contraindications to prophylactic screening of SPTE, on condition that there are abnormalities regarding finger oxygen saturation, quantitative arterial blood gas, or D-dimer assay in DVT patients. In-time examination for SPTE to cut off the “DVT-SPTE-PTE-death” chain is never too careful. We do recommend routine SPTE screening in postoperative neurosurgical patients be included in guideline, yet to be formulated for a balance of benefits and cost. Our recommended screening examinations are finger oxygen saturation, quantitative arterial blood gas, D-dimer assay, and lower limbs ultrasonography, while CTPA can be used in follow-up and prognosis. Therefore, we strongly suggest that we develop a neurosurgical risk-assessment model for SPTE, that is, a neurosurgical-adapted Khorana score, to review SPTE patients at early stage, better within the 1st hour. The indications for early-stage screening of SPTE by a series of reasonable examinations and the routine monitor of D-dimer should be standardized and included in guidelines of neurosurgical patient management, though the interpretation of results depend on many variables and have to be read with caution.

And extra attention should also be attached on patients with dementia, aphasia, who cannot give a history, and who can be even in deep-coma after cranial operations. Actually, such patients are not rare in neurosurgical department, and they are literally “silent” when they suffer PTE. Promptly identification of these patients arise another problem for bed-side physicians and NICU management but will greatly improve the quality of neurosurgical patient care.

## 4 Conclusion

SPTE is dangerous and not rare for neurosurgery patients. Clinicians should be aware of the importance of early recognition of SPTE. Effective management of risk factors of hyper-coagulation state should be the key to prophylaxis. Although the necessity of SPTE routine screening among DVT patients remains controversial, a neurosurgical risk-assessment model for SPTE, that is, a neurosurgical-adapted Khorana Score, should be developed to review SPTE patients at early stage. And routine monitor of D-dimer as well as regular check of lower limbs ultrasonography should be standardized and included in guidelines of neurosurgical patient management.
